# Acute Lung Edema as a Presentation of Severe Acute Reentry High-Altitude Illness in a Pediatric Patient

**DOI:** 10.1155/2020/8871098

**Published:** 2020-08-30

**Authors:** Alfredo Merino-Luna, Julio Vizcarra-Anaya

**Affiliations:** ^1^San Pablo Clinic Huaraz, Emergency and Critical Care Unit, Huaraz, Peru; ^2^Universidad Peruana de Ciencias Aplicadas (UPC), Lima, Peru

## Abstract

Acute high-altitude pulmonary edema (HAPE) is a pathology involving multifactorial triggers that are associated with ascents to altitudes over 2,500 meters above sea level (m). Here, we report two pediatric cases of reentry HAPE, from the city of Huaraz, Peru, located at 3,052 m. The characteristics of both cases were similar, wherein acclimatization to sea level and a subsequent return to the city of origin occurred, and we speculate that it was caused by activation of predisposing factors to HAPE. The diagnosis and management associated with pulmonary hypertension became a determining factor for therapy.

## 1. Introduction

Pathologies associated with changes in barometric pressure are among the most common clinical conditions in countries with high geographical diversity. Hypobaric hypoxia is the trigger for a group of diseases included in what is called high-altitude illness. Acute high-altitude pulmonary edema (HAPE) has been mostly associated with the ascent to places at altitudes over 2,500 meters above sea level (m) [1] and is common among tourists and mountaineers [2]. There are not many cases of HAPE in the pediatric population described in the literature [3, 4]. Furthermore, it has been reported that genetic predisposition may play a critical factor in the development of this syndrome [5], as well as of underlying cardiopulmonary abnormalities [6]. Notably, there are few reported cases of HAPE among highlanders [7]. Below we report two pediatric cases of reentry HAPE that occurred in a native population of Huaraz, a Peruvian city, located at an average altitude of 3,052 m.

## 2. Case Report

**Figure 1:**
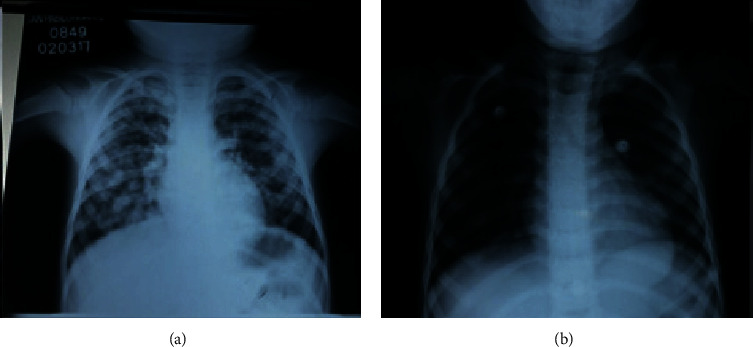
X-ray upon admission to the emergency unit (a) and prior to discharge of the same case (b).

**Figure 2:**
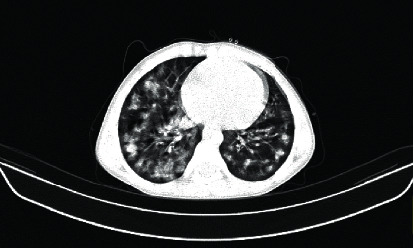
Tomography upon admission to the emergency unit.

A 4-year-old boy from the city of Huaraz (3,052 m) presented with a 2-day sudden onset of the disease, with a progressive course characterized by respiratory difficulty, hemoptoic productive cough, and precordial pain after arrival to the city of Huaraz from the city of Lima. His time of stay in Lima was approximately 13 days and he had a history of being treated for respiratory infection. His vital functions on admission to the emergency unit were as follows:

The following were noted: heart rate = 151 bpm, respiratory rate = 22 breaths/min, blood pressure = 100/60 mmHg, temperature = 36.5°C, and arterial oxygen saturation = 79%. Upon physical examination, subcostal pulses and respiratory effort were evident. Similarly, upon auscultation, there was decreased vesicular murmur in both sides of the hemithorax, and diffuse crackles in both the lung fields. Likewise, a congestive, erythematous oropharynx was evident without plaques or tonsils. Neurologically, the patient was sleepy and scored 15/15 on the Glasgow coma scale. All other laboratory tests were within normal range. Upon admission, he was diagnosed with pneumonia, whereas high-altitude pulmonary edema was ruled out by a score of 7 on the Lake Louise scale. The patient was placed on 4 liters of oxygen through nasal cannula and treatment started with 4 mg of dexamethasone every 6 hours intravenously, 1.4 g of ceftriaxone every 24 hours intravenously, and intensive care unit (ICU) management. On the first day of admission to the ICU, the patient showed ventilatory improvement; therefore, the oxygen volume was reduced to 2 L/min through nasal cannula, and antibiotic treatment was continued upon admission. A cardiology consultation was held, and the patient underwent an echocardiogram that showed moderate pulmonary hypertension with a preserved ejection fraction. On the second day of admission, the patient presented 97% oxygen saturation; consequently, the supplementary oxygen was removed. On the third day of admission to the ICU, the dose of intravenous dexamethasone was reduced to 2 mg every 8 hours, and the patient was discharged from the ICU and moved to the hospitalization floor (Table 1). The patient presented a radiographic film with favorable evolution and clinical improvement on the fifth day of admission Figures 1 and 2 and was subsequently discharged.

## 3. Discussion

Acute high-altitude pulmonary edema (HAPE) has a physiopathology related to genetic predisposing factors affecting voltage-dependent potassium channels, calcium channels, and decreased nitric oxide synthesis [1, 5, 8, 9]. Likewise, an increase in the secretion of inflammatory factors, interleukins, and tumor necrosis factor is evident in these cases [8]. Moreover, HAPE has been linked to polymorphic changes in genes related to the functioning of the renin-angiotensin-aldosterone system [10].

However, this syndrome has been understudied in children, and there are few case reports in which pulmonary hypertension or chromosomal alterations, such as trisomy 21, may have played a preponderant role in this event [3]. On the other hand, there is evidence suggesting that a recent history of respiratory infection increases the risk of developing HAPE [8], and reentry HAPE in this population is more prevalent than in adults [11]. It is in this type of condition that reentry disease and pulmonary hypertension present a significant association. Children born at high altitudes have chronic pulmonary hypertension, which, when travelling to cities at sea level for an extended period of time generates an initial acclimatization that is overexpressed upon return to the area of origin; pulmonary edema may, therefore, be a consequence of vasoconstriction of the pulmonary arteries [12–14].

The study of reentry HAPE and its association with chronic pulmonary hypertension based on highland natives as well as of its physiopathological consequences is recommended. Likewise, the findings of this study point to the importance of evaluating the characteristics of patients who are native to high-altitude areas and of those who are adapted to high altitude.

## Figures and Tables

**Table 1:** Arterial gas and electrolyte analysis.

	Day 1	Day 3
Sodium (mEq/L)	142	136
Potassium (mEq/L)	3.6	3.8
Chlorine (mEq/L)	107.9	106.8
pH	7.38	7.36
PO_2_ (mmHg)	58	67
PCO_2_ (mmHg)	29.2	35
HCO_3_ (mEq/L)	17.5	27
Base excess	−8	−1
Oxygen saturation (%)	90	96
FiO_2_ (%)	21	21
PaFiO_2_	276	319
